# Association of meteorological factors and ambient air pollution on medical care utilization for urolithiasis: a population-based time-series study

**DOI:** 10.1186/s12882-021-02614-5

**Published:** 2021-12-02

**Authors:** Tae Il Noh, Jinwook Hong, Seok Ho Kang, Jaehun Jung

**Affiliations:** 1 Department of Urology, Korea University School of Medicine, Seoul, Republic of Korea; 2 Artificial Intelligence and Big-Data Convergence Center, Gachon University Gil Medical Center, Incheon, Republic of Korea; 3 Department of Preventive Medicine, Gachon University College of Medicine, 38-13, Dokjeom-ro 3, 21565 Incheon, Republic of Korea

**Keywords:** Air pollution, Climate, meteorological factors, Urolithiasis

## Abstract

**Background:**

To identify the association of meteorological factors/ambient air pollutants with medical care utilization for urolithiasis and estimate the effect size/time lags.

**Methods:**

This is a population-based time-series analysis of 300,000 urolithiasis cases from eight large metropolitan areas in Korea. Seventeen meteorological factors and ambient air pollutants were measured daily during 2002–2017 for each metropolis. Data on daily medical utilization owing to urolithiasis were collected. A generalized additive model was used while factoring in the nonlinear relationship between meteorological factors/ambient air pollutants and urolithiasis and a time lag of ≤10 days. A multivariate analysis was performed. Backward elimination with an Akaike information criterion was used for fitting the multivariate model.

**Results:**

Urolithiasis was significantly associated with average temperature, diurnal temperature range, sunshine duration, particulate matter (PM) ≤2.5 μm, and carbon monoxide (CO) levels. The incidence of ureteral stones was positively correlated with average temperature, PM ≤2.5 μm level, and CO level (time lags 0–9, 2–4, and 0–9 days, respectively). The incidence of renal stones was positively correlated with PM ≤2.5 μm and CO levels (time lags 2–4 and 0–9 days, respectively). PM ≤2.5 μm (0.05 and 0.07% per 10 μg/m^3)^ and CO (2.05 and 2.25% per 0.1 ppm) conferred the highest excess risk on ureteral and renal stones.

**Conclusions:**

Urolithiasis is affected by various meteorological factors and ambient air pollutants, PM ≤2.5 μm, and CO levels may be novel potential risk factors for this condition.

**Supplementary Information:**

The online version contains supplementary material available at 10.1186/s12882-021-02614-5.

## Background

Urolithiasis is one of the most prevalent diseases worldwide, and its prevalence is steadily increasing, increasing the socioeconomic burden of diagnosis and treatment [1]. The lifetime prevalence of urolithiasis is reported to range from 6 to 10% in Europe and the United States. Because of the revolutionary advances in the development of endoscopic instruments and techniques, the management of urolithiasis is considered to be relatively easy [2]. Currently, most research on urolithiasis focuses on treatment; however, studies on the prevention and identification of causative factors of urolithiasis are relatively lacking.

Various intrinsic causative factors, such as sex, race, age, mineral metabolism, diet, fluid losses, and dehydration, and even extrinsic factors, such as geographic and meteorological factors, can influence stone formation [3, 4]. Previous studies have reported the influence of seasonal climate on stone formation [5, 6] and the association between ambient temperature and urolithiasis [7–9].

However, to explain the seasonality of urolithiasis with only meteorological factors, substances in the air that are classified as air pollutants may be overlooked. Ambient air pollutants make up the exposed air environment and are influenced by meteorological factors such as wind, humidity, and temperature. Ambient air pollutants such as particulate matter (PM) and carbon monoxide (CO) are considered risk factors for a wide variety of human diseases and affect metabolism [10] by causing inflammation and a reduction in renal function via vascular destruction and oxidative stress [11, 12]. Although these changes may be related to stone formation, the association between urolithiasis and ambient air pollutants has not been explored yet.

Korea has four distinct seasons, and has recorded meteorological factors and air pollutants that match seasonality well. Furthermore, as Korea aims for universal health coverage based on the single-payer system, with the health insurance system covering more than 97% of the population, we can confirm the date the main diagnosis of urolithiasis based on medical care utilization, such as emergency department visits, clinic visit, or hospitalization. These characteristics offer a great opportunity for studying the association between urolithiasis and meteorological factors/ambient air pollutants (MFAPs) [13–15]. This study combined a statistical model with time-series data to investigate the effects of MFAPs on medical care utilization for urolithiasis.

## Methods

### Data acquisition

In Korea, the health insurance system covers more than 97% of the population [9, 16]. A health-care claims database from the National Health Insurance Service, a government-affiliated agency in Korea, was used for this nationwide population-based study. Data on daily urolithiasis incidence between 2002 and 2017 were obtained for eight metropolitan areas: Seoul, Incheon, Daejeon, Gwangju, Daegu, Ulsan, Busan, and Jeju. We defined urolithiasis using the International Classification of Diseases, Tenth Revision, Clinical Modification codes at diagnosis (N20, ureteral stone; N21, renal stone). The medical use for urolithiasis was defined based on each patient’s first hospitalization or outpatient/emergency department visit with the relevant diagnostic code.

**Fig. 1:**
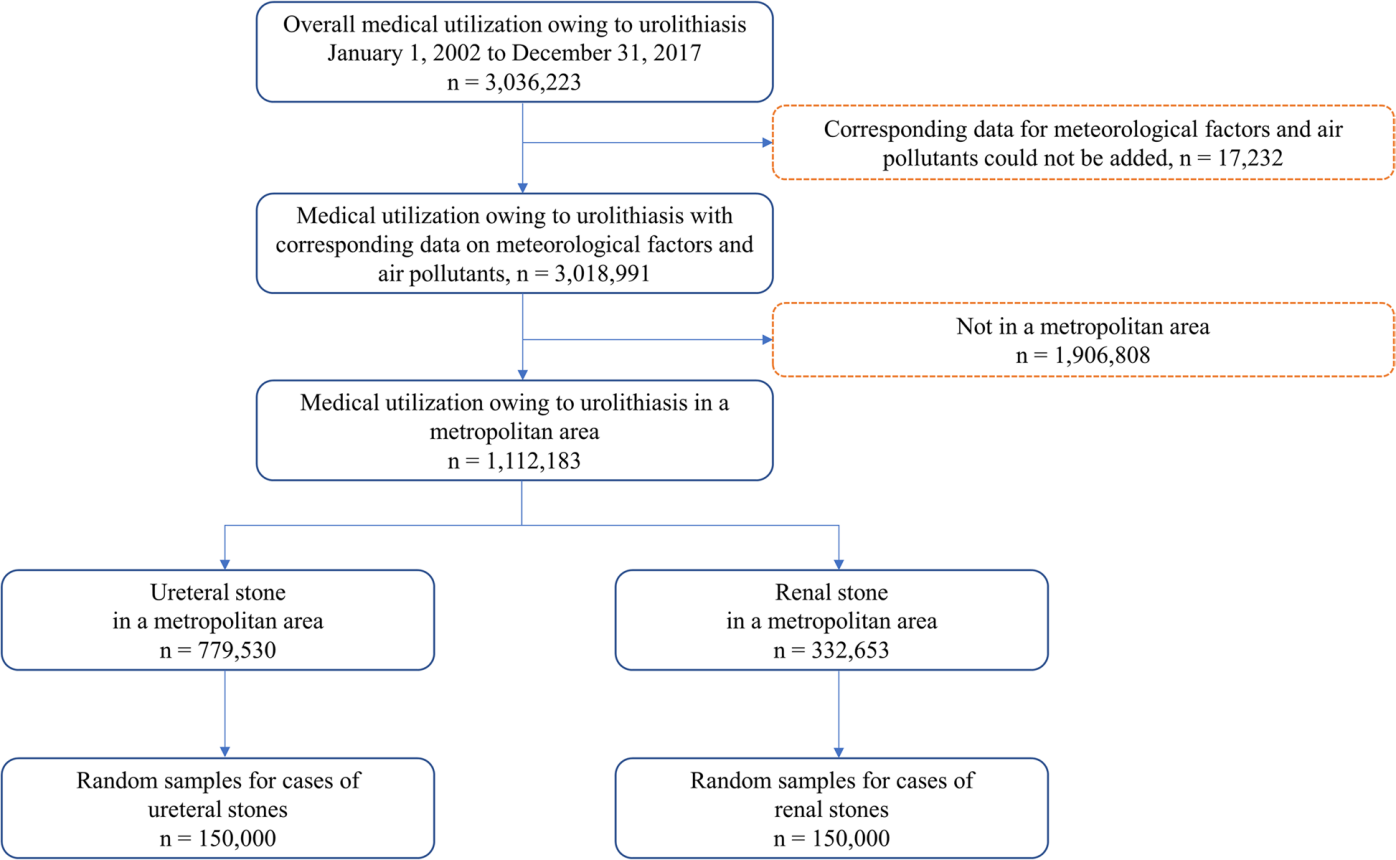
Flowchart of urolithiasis case selection

Between 2002 and 2017, a total of 3,036,223 medical care utilization of urolithiasis were identified. Cases that were not merged with MFAPs (*n* = 17,232) and did not occur in a metropolitan area (n = 1,906,808) were excluded. Renal stones were identified in 332,653 cases, and ureteral stones in 779,530 cases. However, renal and ureteral stones data were too large for nonparametric regression using the generalized additive model (GAM) approach. To overcome these difficulties, we used random sampling with 150,000 cases to reduce the burden of calculation in nonparametric regression (Fig. 1).

Data on meteorological factors, including temperature, humidity, and wind speed, were gathered from the National Climate Data Center through the Korean Meteorological Administration. Information on air pollutants, such as PM ≤2.5 μm in diameter (PM_2.5_), PM ≤10 μm in diameter (PM_10_), CO, nitrogen dioxide, and ozone, was obtained from Air Korea for the same period. As PM_2.5_ has been measured in Korea only since 2015, data on PM_2.5_ before 2015 were treated as missing values.

### Modelling

GAMs [17] are optimized for nonlinear functions and allow for greater flexibility than traditional modelling tools, especially while analysing a time series of weather variables, which generally include time-varying factors that may affect health outcomes. Potential confounders were controlled in our analysis, including trend, seasonality, and day of the week. In the time-series analysis, we considered the partial autocorrelation of the residuals of the Durbin–Watson test model because several meteorological factors are highly correlated, and it was possible to detect time lags. Lag detection was carried out until partial autocorrelation was indicated through white noise. We included those potential confounders as covariates and the sum of the autocorrelation terms in the GAM.

Daily medical care utilization for urolithiasis incidence was considered a count variable, i.e., it followed a Poisson distribution. The overdispersion of urolithiasis incidence was tested before applying Poisson regression in the GAM, following either Poisson or quasi-Poisson analysis. Furthermore, in the Poisson model, we evaluated the urolithiasis incidence in the eight metropolitan areas using the logged variable as the offset variable for controlling the variation in regional occurrence that could affect the incidence.

In the multivariate model, for the selection of meteorological factors, we calculated the Akaike information criterion (AIC) [18] for each candidate factor and compared the AIC values of the models through backward elimination from the Granger causality (GC) test [19]. Since all MFAPs had individual interaction, we need an optimal regression equation along with urolithiasis. Selection aims to reduce or to account for urolithiasis effects. Among all meteorological factors, average temperature (AT), diurnal temperature range (DTR), sunshine duration (SD), PM_2.5_ levels, and CO levels were selected for the model, as they had the lowest AIC values. Our final multivariate model was defined as follows:

$$\log\left[E(Y)\right]=\alpha_0+S\;\left(\mathrm{AT},\mathrm{df}=19\right)+S\;\left(\mathrm{DTR},\mathrm{df}=18\right)+S\;\left(\mathrm{SD},\mathrm{df}=32\right)+S\;\left({\mathrm{PM}}_{2.5},\mathrm{df}=11\right)+S\;\left(\mathrm{CO},\mathrm{df}=4\right)+\mathrm{offset}\;\left(\log\;\left(\mathrm{province}\;\mathrm{population}\right)\right)+\gamma\;\left(\mathrm{day}\;\mathrm{of}\;\mathrm{the}\;\mathrm{week}\right)+\gamma\;\left(\mathrm{year}\right)+{\textstyle\sum_{1\leq\theta\leq j\;}}{\mathrm{AR}}_j,$$

where *E*(*Y*) is the expected daily urolithiasis incidence, *α*_0_ is the intercept, *S* is the smooth function of selected meteorological factors obtained using natural splines, *γ* is the indicator variable for the day of the week and year, and AR_1_,…, AR_j_ are autocorrelation terms. Consequently, in our analysis, the Poisson GAM model with natural splines accounted for no bias in terms of time effects and serial correlation to identify the relationship between the daily urolithiasis incidence and selected meteorological factors.

Statistical analyses were performed using SAS version 9.4 for Windows (SAS Institute, Cary, NC, USA). The results are presented as relative risks (RRs) ratio with 95% confidence intervals (CIs). A *p*-value < 0.05 was considered significant.

## Results

**Fig. 2:**
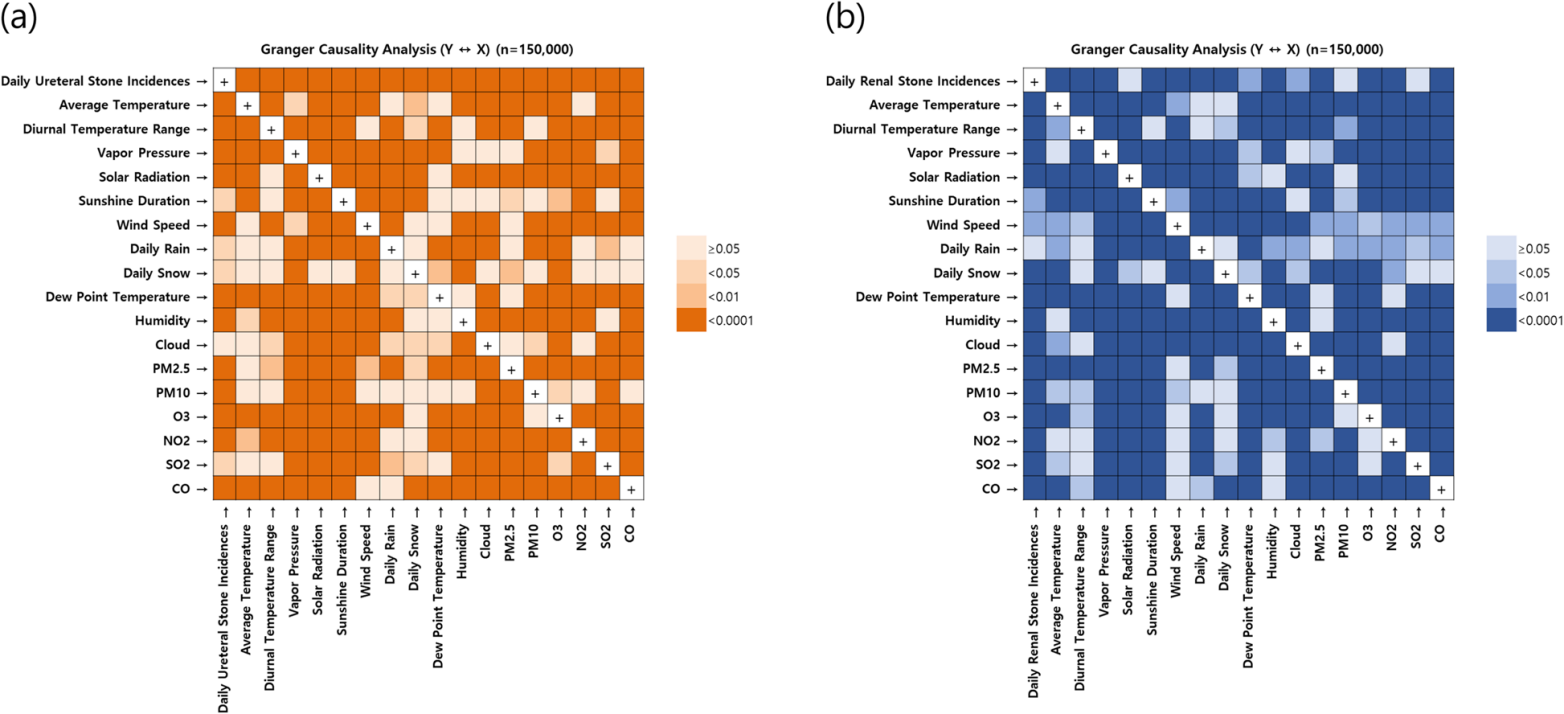
Granger causality graph of the number of medical care utilization for urolithiasis cases and the related MFAPs in Korea: (**a**) ureteral and (**b**) renal stones. The figure shows the direct unilateral and bilateral relationships between urolithiasis and MFAPs. The X- and Y-axes represent the effects and causes, respectively. Therefore, the CO along the Y-axis indicates a cause that can predict the daily urolithiasis medical care utilization. MFAPs, meteorological factors and ambient air pollutants; CO, carbon monoxide

Between 2002 and 2017, the medical care utilization for ureteral and renal stones remained relatively similar (ureteral stones: 142,983 and 166,490 cases; renal stones: 28,973 and 32,021 cases). However, the prevalence of ureteral and renal stones showed a continuous increase from 177,436 to 292,160 cases and from 49,876 to 105,960 cases, respectively. The sex ratio in the medical utilization of urolithiasis was 1.5:1 (men/women: 1.6:1 for ureteral stones and 1.5:1 for renal stones). Those aged 55–69 years had the highest medical care for urolithiasis (Supplementary Fig. 1). Summaries of daily urolithiasis and MFAP data and exposure to meteorological factors have been presented in Supplementary Tables 1–3. The GC tests showed a dynamic correlation between MFAPs and urolithiasis (Fig. 2).

In the univariate GAM, a nonlinear relationship was observed between MFAP and urolithiasis. The AT showed a statistically significant correlation (*p* < 0.0001) with the medical care utilization of urolithiasis. In the most frequently observed interval (interquartile range [IQR]: 10.4 °C–27.2 °C), the risk associated with rising AT constantly increased and showed a linear correlation with the medical care utilization of ureteral (*p* = 0.0007) and renal stones (*p* < 0.001). A positive risk with an inverted U-shape pattern was seen from 22 °C to 32 °C, and the highest risk was found at 26 °C. In addition, there was a variable association with an abrupt decrease in the risk at extreme temperatures (− 10.7 °C to 0.0 °C and > 38 °C) for both ureteral and renal stones. Moreover, there was a significant association between urolithiasis and PM_2.5_ levels (*p* < 0.0001). An excess risk was seen in the most frequently observed interval (IQR: 14.0–38.0 μg/m^3^). In the 0- to 8-μg/m^3^ interval, a stable increase in risk that showed an almost linear association was observed, with the highest risk observed at 8 μg/m^3^. The risk appeared to decrease for values up to 30.0 μg/m^3^. However, considering the observation frequency, it appeared that PM_2.5_ conferred an excess risk in both groups. CO levels showed a statistically significant association with urolithiasis medical care utilization (*p* < 0.0001). A positive risk correlation with an inverted U-shaped pattern was seen in the most frequently observed interval (0.2–1.0 ppm) (Supplementary Fig. 2).

The MFAP combination with the highest fitness was selected as the lowest AIC in the GC test in multivariate analysis. The model that included AT, DTR, SD, and CO and PM_2.5_ levels had the lowest AIC (4.89) (Supplementary Table 4).

**Fig. 3:**
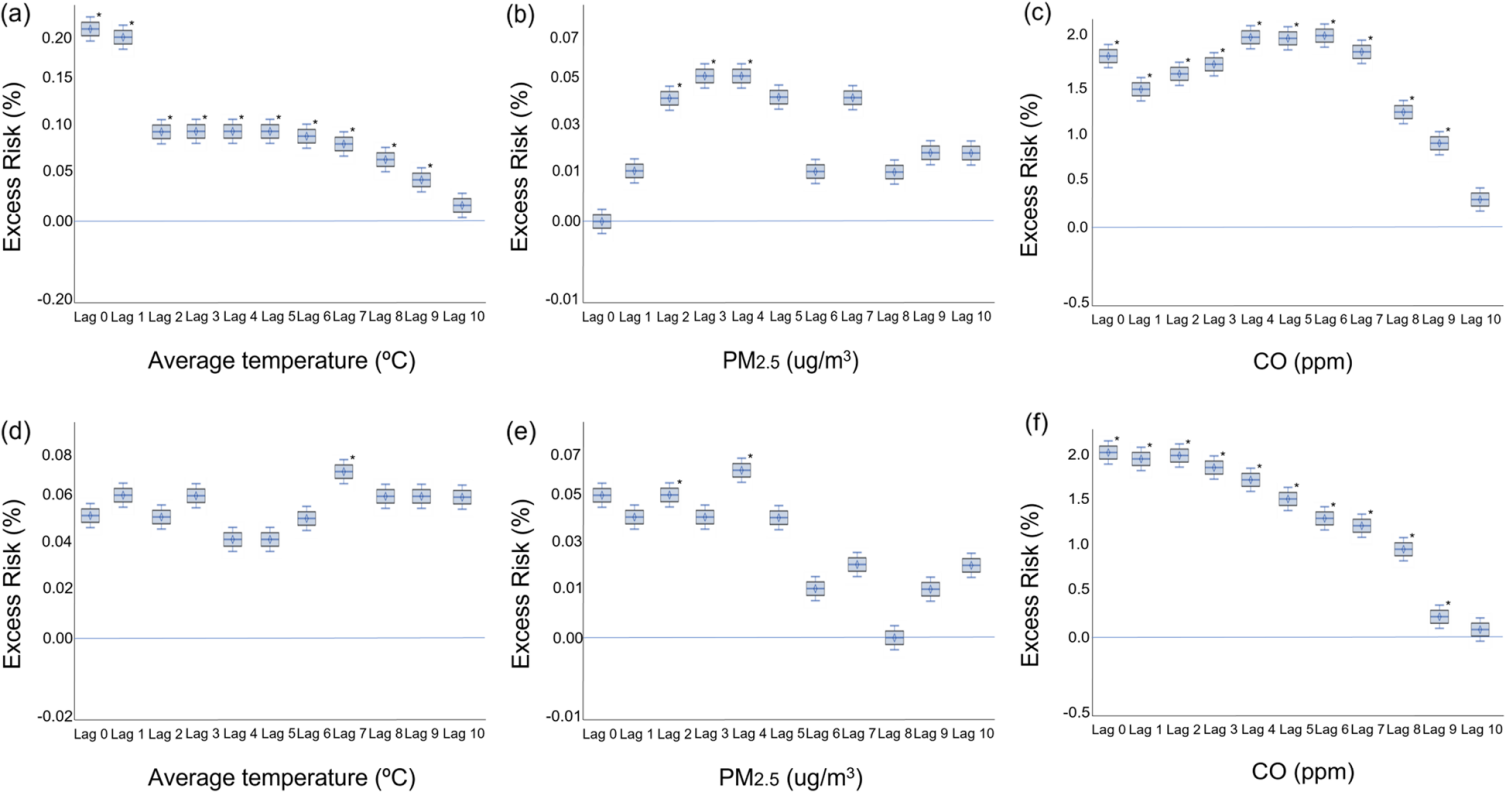
Levels of selected meteorological factors and ambient air pollutants and the adjusted excess risk of medical care utilization for urolithiasis. Ureteral stones: (**a**) average temperature, (**b**) PM2.5, and (**c**) CO. Renal stones: (**d**) average temperature, (**e**) PM2.5, and (**f**) CO. PM2.5, particulate matter ≤2.5 μm; CO, carbon monoxide. The X-axis shows lag days. The Y-axis shows the percentage of adjusted excess risk with 95% confidence intervals. *p < 0.05

The multivariate analysis provided the time lag for the effect of the MFAP on the risk in each period. The association between AT and urolithiasis was different for ureteral and renal stones. An increase in AT reflected a significant increase in the risk of ureteral stones until 9 days later, but the effect for renal stones was not significant. PM_2.5_ levels showed a significantly positive association 2–4 days before the occurrence of ureteral stones, and a similar pattern was observed for renal stones. The excess risk was 0.05–0.07% per 10-μg/m^3^ increase in PM_2.5_ levels. CO levels were positively correlated with urolithiasis medical care utilization over 9 days, and the excess risk was up to 2.25% per 0.1-ppm increase in CO levels (Fig. 3; Table 1).

**Table 1:** Generalized additive model (GAM) with cubic spline analysis for daily urolithiasis incidence between 2002 and 2017 according to time-lags

Time	Variables	Ureteral Stone							
		Univariate				Multivariate			
		RR	ER(%)	95% CI	P-value	RR	ER(%)	95% CI	*P*-value
Lag 0	Avg. Temp.	1.0005	0.05	[1.0002–1.0035]	0.0007	1.0021	0.21	[1.0012–1.0031]	<.0001
	PM_2.5_	1.0041	0.41	[1.0039–1.0068]	<.0001	1.0000	0.00	[0.9996–1.0004]	0.8664
	CO.	1.0150	1.50	[1.0137–1.0290]	<.0001	1.0171	1.71	[1.0157–1.0185]	<.0001
Lag 1	Avg. Temp.	1.0005	0.05	[1.0002–1.0008]	0.0009	1.0020	0.20	[1.0011–1.0029]	<.0001
	PM_2.5_	1.0002	0.02	[0.9998–1.0006]	0.2517	1.0001	0.01	[0.9997–1.0006]	0.4984
	CO.	1.0123	1.23	[1.0109–1.0138]	<.0001	1.0151	1.51	[1.0136–1.0165]	<.0001
Lag 2	Avg. Temp.	1.0004	0.04	[1.0002–1.0008]	0.0012	1.0009	0.09	[1.0003–1.0014]	0.0016
	PM_2.5_	1.0000	0.00	[0.9996–1.0004]	0.9435	1.0004	0.04	[1.0000–1.0009]	0.0343
	CO.	1.0148	1.48	[1.0101–1.0129]	<.0001	1.0167	1.67	[1.0151–1.0183]	<.0001
Lag 3	Avg. Temp.	1.0005	0.05	[1.0002–1.0008]	0.0008	1.0009	0.09	[1.0003–1.0014]	0.0017
	PM_2.5_	1.0001	0.01	[0.9997–1.0005]	0.6229	1.0005	0.05	[1.0001–1.0009]	0.0137
	CO.	1.0124	1.24	[1.0110–1.0139]	<.0001	1.0172	1.72	[1.0156–1.0188]	<.0001
Lag 4	Avg. Temp.	1.0005	0.05	[1.0003–1.0008]	0.0003	1.0009	0.09	[1.0004–1.0014]	0.0011
	PM_2.5_	1.0000	0.00	[0.9997–1.0005]	0.7510	1.0005	0.05	[1.0001–1.0009]	0.0253
	CO.	1.0133	1.33	[1.0119–1.0148]	<.0001	1.0188	1.88	[1.0172–1.0204]	<.0001
Lag 5	Avg. Temp.	1.0005	0.05	[1.0002–1.0008]	0.0007	1.0009	0.09	[1.0004–1.0014]	0.0009
	PM_2.5_	1.0000	0.00	[0.9996–1.0005]	0.7953	1.0004	0.04	[0.9999–1.0008]	0.1022
	CO.	1.0132	1.32	[1.0118–1.0147]	<.0001	1.0187	1.87	[1.0171–1.0203]	<.0001
Lag 6	Avg. Temp.	1.0004	0.04	[1.0002–1.0008]	0.0013	1.0008	0.08	[1.0003–1.0014]	0.0027
	PM_2.5_	1.0002	0.02	[0.9998–1.0006]	0.3182	1.0001	0.01	[0.9997–1.0005]	0.5135
	CO.	1.0135	1.35	[1.0122–1.0150]	<.0001	1.0189	1.89	[1.0173–1.0204]	<.0001
Lag 7	Avg. Temp.	1.0004	0.04	[1.0002–1.0008]	0.0023	1.0007	0.07	[1.0004–1.0010]	0.0096
	PM_2.5_	1.0000	0.00	[0.9997–1.0005]	0.7174	1.0004	0.04	[1.0000–1.0008]	0.0805
	CO.	1.0135	1.35	[1.0121–1.0149]	<.0001	1.0178	1.78	[1.0162–1.0193]	<.0001
Lag 8	Avg. Temp.	1.0004	0.04	[1.0002–1.0008]	0.0019	1.0006	0.06	[1.0002–1.0009]	0.0177
	PM_2.5_	1.0003	0.03	[0.9999–1.0007]	0.1321	1.0001	0.01	[0.9997–1.0005]	0.6095
	CO.	1.0124	1.24	[1.0110–1.0138]	<.0001	1.0127	1.27	[1.0111–1.0143]	<.0001
Lag 9	Avg. Temp.	1.0004	0.04	[1.0001–1.0007]	0.0248	1.0004	0.04	[1.0001–1.0008]	0.0098
	PM_2.5_	1.0002	0.02	[0.9998–1.0007]	0.2368	1.0002	0.02	[0.9997–1.0006]	0.4346
	CO.	1.0090	0.90	[1.0074–1.0107]	<.0001	1.0087	0.87	[1.0071–1.0102]	<.0001
Lag 10	Avg. Temp.	1.0004	0.04	[0.9999–1.0009]	0.0668	1.0002	0.02	[0.9999–1.0005]	0.1505
	PM_2.5_	1.0002	0.02	[0.9998–1.0006]	0.3368	1.0002	0.02	[0.9998–1.0006]	0.3989
	CO.	1.0020	0.20	[1.0000–1.0041]	0.0445	1.0037	0.37	[0.9998–1.0076]	0.2551
Time	Variables	Renal Stone							
		Univariate				Multivariate			
		RR	ER(%)	95% CI	P-value	RR	ER(%)	95% CI	P-value
Lag 0	Avg. Temp.	1.0027	0.27	[1.0015–1.0144]	<.0001	1.0005	0.05	[0.9998–1.0071]	0.1425
	PM_2.5_	1.0004	0.04	[0.9999–1.0054]	0.1236	1.0005	0.05	[1.0000–1.0055]	0.0488
	CO.	1.0163	1.63	[1.0147–1.0318]	<.0001	1.0205	2.05	[1.0187–1.0381]	<.0001
Lag 1	Avg. Temp.	1.0026	0.26	[1.0015–1.0038]	<.0001	1.0006	0.06	[0.9999–1.0012]	0.0936
	PM_2.5_	1.0004	0.04	[0.9999–1.0009]	0.1524	1.0004	0.04	[0.9999–1.0009]	0.1027
	CO.	1.0162	1.62	[1.0146–1.0178]	<.0001	1.0190	1.90	[1.0172–1.0208]	<.0001
Lag 2	Avg. Temp.	1.0025	0.25	[1.0013–1.0037]	<.0001	1.0005	0.05	[0.9998–1.0012]	0.1425
	PM_2.5_	1.0004	0.04	[0.9999–1.0009]	0.1226	1.0005	0.05	[1.0000–1.0010]	0.0488
	CO.	1.0152	1.52	[1.0136–1.0167]	<.0001	1.0195	1.95	[1.0179–1.0210]	<.0001
Lag 3	Avg. Temp.	1.0025	0.25	[1.0013–1.0036]	<.0001	1.0006	0.06	[0.9999–1.0012]	0.0897
	PM_2.5_	1.0004	0.04	[0.9999–1.0009]	0.1345	1.0004	0.04	[0.9999–1.0009]	0.0964
	CO.	1.0145	1.45	[1.0129–1.0161]	<.0001	1.0175	1.75	[1.0158–1.0193]	<.0001
Lag 4	Avg. Temp.	1.0020	0.20	[1.0008–1.0031]	0.0010	1.0004	0.04	[0.9998–1.0011]	0.2062
	PM_2.5_	1.0005	0.05	[1.0000–1.0010]	0.0551	1.0006	0.06	[1.0001–1.0011]	0.0195
	CO.	1.0157	1.57	[1.0141–1.0172]	<.0001	1.0165	1.65	[1.0149–1.0181]	<.0001
Lag 5	Avg. Temp.	1.0019	0.19	[1.0007–1.0030]	0.0013	1.0004	0.04	[0.9997–1.0011]	0.2333
	PM_2.5_	1.0004	0.04	[0.9999–1.0009]	0.1392	1.0004	0.04	[0.9999–1.0009]	0.0957
	CO.	1.0156	1.56	[1.0140–1.0172]	<.0001	1.0152	1.52	[1.0134–1.0170]	<.0001
Lag 6	Avg. Temp.	1.0020	0.20	[1.0008–1.0031]	0.0008	1.0005	0.05	[0.9998–1.0012]	0.1316
	PM_2.5_	1.0001	0.01	[0.9996–1.0006]	0.6866	1.0001	0.01	[0.9996–1.0006]	0.8084
	CO.	1.0149	1.49	[1.0133–1.0165]	<.0001	1.0136	1.36	[1.0122–1.0150]	<.0001
Lag 7	Avg. Temp.	1.0026	0.26	[1.0014–1.0037]	<.0001	1.0007	0.07	[1.0001–1.0014]	0.0314
	PM_2.5_	1.0004	0.04	[0.9999–1.0009]	0.1535	1.0002	0.02	[0.9997–1.0007]	0.4583
	CO.	1.0117	1.17	[1.0101–1.0133]	<.0001	1.0124	1.24	[1.0106–1.0142]	<.0001
Lag 8	Avg. Temp.	1.0026	0.26	[1.0015–1.0038]	<.0001	1.0006	0.06	[1.0000–1.0013]	0.0617
	PM_2.5_	1.0001	0.01	[0.9996–1.0007]	0.5666	1.0000	0.00	[0.9995–1.0005]	0.9405
	CO.	1.0096	0.96	[1.0080–1.0111]	<.0001	1.0091	0.91	[1.0077–1.0105]	<.0001
Lag 9	Avg. Temp.	1.0018	0.18	[1.0006–1.0030]	0.0325	1.0006	0.06	[1.0000–1.0013]	0.0608
	PM_2.5_	1.0002	0.02	[0.9997–1.0007]	0.4270	1.0001	0.01	[0.9996–1.0006]	0.7988
	CO.	1.0058	0.58	[1.0042–1.0074]	<.0001	1.0038	0.38	[1.0028–1.0048]	<.0001
Lag 10	Avg. Temp.	1.0002	0.02	[0.9998–1.0005]	0.0710	1.0006	0.06	[1.0000–1.0013]	0.0581
	PM_2.5_	1.0000	0.00	[0.9995–1.0005]	0.9853	1.0002	0.02	[0.9997–1.0008]	0.3878
	CO.	1.0014	0.14	[0.9998–1.0030]	0.1138	1.0011	0.11	[0.9999–1.0022]	0.0874

*Avg. Temp*. Average Temperature, *RR* relative risks, *CI* confidence intervals

## Discussion

We examined the effects of MFAPs on urolithiasis in our study. Our results demonstrated a consistent correlation between urolithiasis and meteorological factors (AT, DTR, and SD) and identified PM_2.5_ and CO levels as novel potential risk factors for this condition.

In the univariate GAM, a nonlinear significant correlation was observed between MFAP and urolithiasis. Exposure to PM_2.5_ showed a typical significant inverted U-shape correlation at a concentration of 0–30 μg/m^3^. Moreover, the association appeared to be less relevant at concentrations > 30 μg/m^3^, where PM is classified as a potential risk factor for carcinogenesis and several other diseases of great concern to public health owing to its toxicity [20]. However, these data may reflect the preventive effect of reducing outdoor activity, and masks or air purifiers use through national notifications depending on the air pollutants concentration. The multivariate analysis demonstrated the effect size and time lag for the impact of the MFAP and the risk in each period. PM2.5 levels showed a significant association for ureteral stones after 2–4 days, with the excess risk being 0.05–0.07% per 10-μg/m^3^ increase, and PM_2.5_ levels had a subacute effect on urolithiasis. For renal stones, PM_2.5_ showed excess risk with a sporadic lag day-dependent pattern. However, the concentration of PM_2.5_ from days 2 to 4 showed a significant association, and the excess risk was 0.05–0.07% per 10-μg/m^3^ increase in levels.

PM inhaled through the nasal cavity and lungs can reach the alveoli if the particle size is ≤10 μm, and smaller PMs can even penetrate deeper. PMs smaller than 1 μm can enter the circulatory system, similar to gas molecules, and reach the kidneys via the bloodstream. They can directly or indirectly affect profile changes in renal function and urine metabolites [21, 22]. Exposure to PM can cause vascular damage and oxidative stress, which, in turn, causes inflammation and direct membrane damage to the kidneys, leading to membranous nephropathy, renal function degradation (reduced estimated glomerular filtration rate), and subsequent abnormalities in urinary metabolite profiles/reduced citrate levels [11, 12, 23, 24]. But considering that the effect of PM_2.5_ showed a lag time of 2–5 days, stone attack may be accelerated or influenced by the serial changes in urine metabolite profiles or urinary tract constriction due to PM exposure rather than stone formation.

In the univariate analysis, CO levels showed the highest risk association at 0.55 ppm for ureteral stones and 0.58 ppm for renal stones. A continuous risk relationship up to 1.0 ppm; a decrease in risk was observed after 1.0 ppm. As for PM_2.5_, these data could also reflect the previously mentioned preventive effects. CO levels were continuously correlated with urolithiasis over 9 days without time lag, and the excess risk was up to 2.25% per 0.1 ppm.

CO has an immunosuppressive effect on the human body, which may result from acute or chronic toxicity [25]. CO can cause inflammation and immunosuppression, which may accelerate or influence stone attack.

Our study has several strengths. First, it was the first to investigate the influence of variable MFAPs on urolithiasis. Second, we analyzed national-level data from the Korea National Health Insurance Services, and our sample size (150,000) was much larger than that of other studies. Further, our study encompassed the capital and seven other areas in Korea, which minimized the effect of region-specific variables, such as race, culture, socioeconomics, and climate. Third, through a time-series multilevel approach based on Poisson analysis with GAM after GC testing, a high-accuracy statistical method, we analysed the interactions among all 13 MFAPs described by the Korean Meteorological Agency. Thus, reflecting the real-world effects and interactions of PM_2.5_ and CO with various meteorological factors.

However, several limitations of this study must also be acknowledged. First, we sampled patients who lived in metropolitan cities. Therefore, individual lifestyles were not considered, and we assumed that these individuals were exposed to the same environment. Thus, the possibility of an ecological fallacy cannot be ruled out. Second, confounding factors, such as intrinsic and extrinsic factors affecting stone formation, were not considered. Several pathophysiologic derangements contribute to stone formation. These are either alone or combined with confounding factors, including the genetic factors, occupation, obesity, underlying diseases such as metabolic syndrome, diabetes, and cardiovascular disease, individual lifestyle, and dietary habits including salt consumption, beverages, and the hardness and mineral compositions in water and amount of water intake [26–28]. Because of these multiple causes associated with stone formation, understanding the underlying genetic factors, metabolic disorders, and environmental factors that predispose to stone formation is required. However, we could not consider all multiple factors in this study, which may have introduced bias.

The results of the multivariate analysis illustrated that the analysis of meteorological factors after adjusting for temperature, PM_2.5_, and CO provides an ambiguous interpretation of the lag day. These further restrict the analysis conducted among patients who have visited the emergency department or clinic or were hospitalized due to stone-induced symptoms, such as acute pain and haematuria. Painless, undiagnosed, and self-resolved stones were not included, and there may be a gap between stone formation and the onset of symptoms. However, Wimpissinger et al. reported that only 1.1% of patients had an asymptomatic stone in their investigation [29]. Therefore, the influence of the minor multiple compounding factors not included in this study may be insignificant.

Additionally, chronic urolithiasis involving renal stones shows a lower association with meteorological factors because symptoms, such as pain, may be identified only at the time of examination. Finally, the risks associated with air pollutants may be underestimated. They might reflect the preventive effects of reduced outdoor activity owing to national notifications and the use of masks or air purifiers, depending on the concentration of air pollutants.

## Conclusions

Urolithiasis is affected by various MFAPs. In addition to seasonal variation (AT, DTR, and SD), PM_2.5_ and CO appear to be novel potential risk factors for urolithiasis. These can act as important regulatory factors for disease course, and urolithiasis prevention can be mediated through national notifications and programs. In the future, more confirmatory studies and improved public awareness regarding these air pollutants are needed for the clinical prevention and management of urolithiasis.

## Supplementary Information

**Additional file 1: Supplementary Table 1**. Summary of the daily incidence of urolithiasis and data on meteorological factors and ambient air pollutants in Korea between 2002 and 2017. **Supplementary Table 2** Summary statistics for random sampling of urolithiasis cases with corresponding data on meteorological factors and ambient air pollutants in Korea between 2002 and 2017. **Supplementary Table 3**. Comparison of urolithiasis incidence rates among different age groups between 2002 and 2017. **Supplementary Table 4**. Akaike information criterion for the association between daily medical care utilization for urolithiasis and selected meteorological factors and ambient air pollutants (MFAPs). **Supplementary Figure 1** Prevalence and incidence of (a) ureteral and (b) renal stones between 2002 and 2017. (c) Histogram of the sex-dependent incidence of urolithiasis according to age group. **Supplementary Figure 2**. Generalized additive model with natural splines for the effect of selected MFAPs on the number of medical care utilization for urolithiasis. Ureteral stones: (a) average temperature, (b) PM2.5, and (c) CO. Renal stones: (d) average temperature, (e) PM2.5, and (f) CO. The bold line indicates the relative effect sizes for urolithiasis, and the blue area indicates 95% confidence intervals. The X- and Y-axes represent the selected MFAPs and relative effect sizes for urolithiasis, respectively. MFAPs, meteorological factors and ambient air pollutants; PM2.5, particulate matter ≤2.5 μm; CO, carbon monoxide.

## Data Availability

The datasets generated during and/or analysed during the current study are available from the corresponding author on reasonable request.

## Abbreviations

AIC: Akaike information criterion
AT: Average temperature
CIs: Confidence intervals
CO: Carbon monoxide
DTR: Diurnal temperature range
GAMs: Generalized additive models
GC: Granger causality
IQR: Interquartile range
MFAPs: Meteorological factors/ambient air pollutants
PM: Particulate matter
PM_2.5_: PM ≤2.5 μm in diameter
PM_10_: PM ≤10 μm in diameter
RRs: Relative risks
SD: Sunshine duration
