# Supplementary material for: Association of meteorological factors and ambient air pollution on medical care utilization for urolithiasis: a population-based time-series study
Source: BMC Nephrol. 2021 Dec 2;22:402. doi: 10.1186/s12882-021-02614-5 (PMC8638132; doi:10.1186/s12882-021-02614-5)
Supplement: Supplementary file 1 — Additional file 1: Supplementary Table 1. Summary of the daily incidence of urolithiasis and data on meteorological factors and ambient air pollutants in Korea between 2002 and 2017. Supplementary Table 2 Summary statistics for random sampling of urolithiasis cases with corresponding data on meteorological factors and ambient air pollutants in Korea between 2002 and 2017. Supplementary Table 3. Comparison of urolithiasis incidence rates among different age groups between 2002 and 2017. Supplementary Table 4. Akaike information criterion for the association between daily medical care utilization for urolithiasis and selected meteorological factors and ambient air pollutants (MFAPs). Supplementary Figure 1 Prevalence and incidence of (a) ureteral and (b) renal stones between 2002 and 2017. (c) Histogram of the sex-dependent incidence of urolithiasis according to age group. Supplementary Figure 2. Generalized additive model with natural splines for the effect of selected MFAPs on the number of medical care utilization for urolithiasis. Ureteral stones: (a) average temperature, (b) PM2.5, and (c) CO. Renal stones: (d) average temperature, (e) PM2.5, and (f) CO. The bold line indicates the relative effect sizes for urolithiasis, and the blue area indicates 95% confidence intervals. The X- and Y-axes represent the selected MFAPs and relative effect sizes for urolithiasis, respectively. MFAPs, meteorological factors and ambient air pollutants; PM2.5, particulate matter ≤2.5 μm; CO, carbon monoxide. [file 12882_2021_2614_MOESM1_ESM.pdf]

Supplementary Information

Effect of meteorological factors and ambient air pollution on urolithiasis: a population-based time-series study

Content

Supplementary Table 1

Summary of the daily incidence of urolithiasis and data on meteorological factors and ambient air pollutants in Korea between 2002 and 2017

Supplementary Table 2

Summary statistics for random sampling of urolithiasis cases with corresponding data on meteorological factors and ambient air pollutants in Korea between 2002 and 2017

Supplementary Table 3

Comparison of urolithiasis incidence rates among different age groups between 2002 and 2017

Supplementary Table 4. Akaike information criterion for the association between daily medical care utilization for urolithiasis and selected meteorological factors and ambient air pollutants (MFAPs)

Supplementary Figure 1

Prevalence and incidence of (a) ureteral and (b) renal stones between 2002 and 2017. (c) Histogram of the sex-dependent incidence of urolithiasis according to age group.

Supplementary Figure 2

Generalized additive model with natural splines for the effect of selected MFAPs on the number of medical care utilization for urolithiasis. Ureteral stones: (a) average temperature, (b) $PM_{2.5}$, and (c) CO. Renal stones: (d) average temperature, (e) $PM_{2.5}$, and (f) CO. The bold line indicates the relative effect sizes for urolithiasis, and the blue area indicates 95% confidence intervals. The X- and Y-axes represent the selected MFAPs and relative effect sizes for urolithiasis, respectively. MFAPs, meteorological factors and ambient air pollutants; $PM_{2.5}$, particulate matter ≤ 2.5 µm; CO, carbon monoxide.

Supplementary Table 1

Summary of the daily incidence of urolithiasis and data on meteorological factors and ambient air pollutants in Korea between 2002 and 2017

		2002	2003	2004	2005	2006	2007	2008	2009	2010	2011	2012	2013	2014	2015	2016	2017
**Urolithiasis (*N* = 3,036,223)**		171,956	172,797	174,288	181,911	183,137	195,070	194,670	196,933	193,521	194,440	198,574	198,705	191,282	194,439	195,998	198,511
**Incidence**																	
Ureteral Stone (n = 2,546,196)		142,983	143,410	144,748	152,237	153,120	162,972	163,117	165,286	164,120	163,422	166,995	167,894	161,117	163,542	164,743	166,490
Renal Stone (n = 490,027)		28,973	29,378	29,540	29,674	30,017	32,098	31,553	31,647	29,401	31,018	31,579	30,811	30,165	30,897	31,255	32,021
**Gender**																	
Ureteral Stone																	
Male		91,396	91,536	94,419	100,165	105,669	107,544	107,899	110,377	108,966	109,109	111,994	113,192	109,411	110,658	111,477	112,577
Female		51,587	51,874	50,329	52,072	52,451	55,428	55,218	54,909	55,154	54,313	55,001	54,702	51,706	52,884	53,266	53,913
Renal Stone																	
Male		17,152	17,047	17,631	17,797	18,005	19,098	18,910	19,171	17,908	18,757	19,074	18,607	18,241	18,748	18,696	18,950
Female		11,821	12,331	11,909	11,877	12,012	13,000	12,643	12,476	11,493	12,261	12,505	12,204	11,924	12,149	12,559	13,071
**Age, Year**																	
Ureteral Stone	< 20	4,238	3,962	3,811	3,781	3,790	3,970	3,905	3,670	3,719	3,470	2,962	3,176	2,838	2,320	2,422	2,669
	20 - 60	117,089	116,738	117,771	123,396	122,428	126,069	125,232	125,184	122,214	121,148	120,437	120,088	112,205	112,479	110,609	109,472
	> 60	21,656	22,710	23,166	25,060	26,902	32,933	33,980	36,432	38,187	38,804	43,596	44,630	46,074	48,743	51,712	54,349
Renal Stone	< 20	876	880	893	832	849	899	857	899	824	894	897	841	822	758	848	844
	20 - 60	22,426	22,786	23,023	23,342	23,669	25,993	25,441	25,436	23,502	24,792	25,407	24,919	23,918	24,811	24,610	25,027
	> 60	5,671	5,712	5,624	5,500	5,499	5,206	5,255	5,312	5,075	5,332	5,275	5,051	5,425	5,328	5,797	6,150
**Meteorology (Mean, SD)**																	
Average Temperature (°C)		12.3(9.6)	14.1(8.8)	14.4(9.4)	13(10.3)	14.1(9.3)	14.9(9.0)	13.9(9.6)	13.9(9.4)	13.4(9.6)	12.8(9.4)	12.9(9.8)	13.6(9.6)	14.1(9.6)	14.0(9.4)	14.3(9.2)	14.0(9.3)
High Temperature (°C)		16.7(9.9)	18.5(9.0)	19.1(9.5)	17(10.4)	18.5(9.5)	19.1(8.9)	18.4(9.8)	18.4(9.6)	17.8(9.7)	17.2(9.5)	17.4(9.9)	18.1(9.7)	18.9(9.7)	18.9(9.6)	19.0(9.3)	18.8(9.3)
Low Temperature (°C)		8.5(9.7)	10.3(9.1)	10.4(9.6)	9.6(10.5)	10.4(9.5)	11.3(9.3)	10.1(9.7)	10.1(9.5)	9.8(10.9)	9.2(10.6)	9.1(11.0)	9.8(9.9)	10.2(9.9)	10.0(9.5)	10.3(9.4)	9.9(9.6)
Daily Range (°C)		8.2(3.0)	8.1(3.0)	8.7(3.1)	8.3(2.8)	8.1(2.9)	7.8(2.9)	8.3(2.9)	8.3(3.0)	7.9(2.9)	8.0(3.0)	8.3(2.7)	8.2(3.1)	8.6(3.1)	8.8(3.2)	8.7(2.9)	8.8(3.0)
Vapor Pressure (hPa)		11.1(7.7)	12.6(7.6)	12.5(8.1)	12.1(8.6)	12.3(7.9)	13.2(8.3)	11.9(7.8)	11.7(7.4)	12.5(8.9)	11.4(8.3)	11.3(8.3)	12.3(8.8)	12.9(8.4)	12.3(7.8)	12.9(8.8)	12.3(8.9)
Solar Radiation (MJ/m^2)		12.5(6.8)	12.1(6.7)	13.1(6.9)	13.4(7.1)	12.8(6.8)	12.5(6.8)	13.5(6.8)	13.8(7.1)	12.9(6.7)	13.1(6.9)	12.8(6.4)	13.0(6.8)	12.9(6.3)	13.1(6.6)	13.2(6.4)	13.7(6.9)

Sunshine Duration (hr)	12.0(1.7)	12.3(1.7)	12.2(1.7)	12.2(1.7)	12.2(1.7)	12.2(1.7)	12.2(1.7)	12.2(1.7)	12.2(1.7)	12.1(1.7)	12.2(1.7)	12.2(1.7)	12.2(1.7)	12.1(1.7)	12.2(1.7)	12.2(1.7)
Wind Speed (m/s)	2.4(1.2)	2.1(0.9)	2.3(0.9)	2.5(1.0)	2.3(1.0)	2.4(1.0)	2.4(0.9)	2.5(1.0)	2.5(1.0)	2.6(1.0)	2.7(1.0)	2.7(1.1)	2.5(1.0)	2.6(1.0)	2.4(1.0)	2.3(0.9)
Daily Rain (mm)	10(24.2)	14(26.7)	10(18.7)	9.8(20.2)	9.4(23.7)	7.9(14.0)	8.8(18.8)	13(31.5)	10(19.9)	12.8(31)	10.8(21)	9.5(18.1)	8.3(16.7)	6.1(10.6)	7.9(16.9)	6.2(16.3)
Dew Point Temperature (°C)	4.8(11.3)	7.3(10.6)	6.6(11.3)	5.4(12.6)	6.5(11.2)	7.7(10.7)	5.9(11.6)	5.8(11.2)	6.1(12.2)	4.6(12.5)	4.4(12.5)	5.8(12.1)	7.1(11.7)	6.8(10.6)	6.8(12.0)	5.8(12.3)
Humidity (%)	63(15.5)	65(15.3)	62(15.4)	61(15.9)	62(15.4)	64(14.6)	61(15.2)	61(15.8)	64(15.6)	60(16.9)	59(16.1)	62(16.1)	65(16.9)	65(16.2)	64(16.4)	61(16.1)
Daily snow (cm)	1.4(1.5)	2.1(2.2)	3.8(5.4)	3.5(6.4)	3.9(3.1)	1.4(2.6)	3.7(3.0)	2.4(2.2)	8.2(6.8)	2.7(2.1)	3.6(2.9)	5.5(4.3)	2.1(1.8)	2.4(2.5)	1.9(2.9)	1.9(1.6)
Cloud (1/10)	4.8(3.3)	5.4(3.2)	4.5(3.1)	4.5(3.2)	5.1(3.0)	5.2(3.1)	4.7(3.0)	4.6(3.1)	5.1(3.2)	5.0(3.3)	4.9(3.0)	4.8(3.1)	4.8(3.1)	4.8(3.1)	4.8(2.9)	4.6(3.0)
**Air Pollutants (Mean, SD)**																
$PM_{2.5}$ (ug/m^3)	-	-	-	-	-	-	-	-	-	-	28(12)	27(13)	25(14)	24(12)	25(14)	23(12)
PM_{10} (ug/m^3)	72(82)	62(34)	59(27)	58(27)	57(36)	57(31)	56(30)	54(29)	50(28)	49(30)	45(21)	47(23)	48(26)	47(34)	46(19)	45(20)
O_3 (100ppb)	1.5(0.8)	1.6(0.8)	1.7(0.9)	1.8(0.9)	1.8(0.9)	1.8(0.9)	1.9(0.9)	2.1(1.0)	1.9(0.9)	1.9(1.0)	2.1(1.0)	2.2(1.0)	2.3(1.1)	2.2(1.0)	2.4(1.1)	2.5(1.1)
NO_2 (100ppb)	3.3(1.1)	3.3(1.3)	3.2(1.2)	3.1(1.2)	3.2(1.2)	3.3(1.4)	3.4(1.4)	3.3(1.3)	3.2(1.3)	3.2(1.3)	3.1(1.2)	3.2(1.3)	3.1(1.3)	3.1(1.3)	2.9(1.1)	2.8(1.1)
SO_2 (100ppb)	0.5(0.2)	0.5(0.2)	0.5(0.2)	0.5(0.2)	0.5(0.2)	0.6(0.3)	0.6(0.2)	0.5(0.2)	0.5(0.2)	0.5(0.2)	0.5(0.2)	0.5(0.2)	0.5(0.2)	0.5(0.1)	0.4(0.1)	0.4(0.1)
CO (10ppm)	0.7(0.2)	0.6(0.2)	0.6(0.2)	0.6(0.2)	0.6(0.2)	0.6(0.3)	0.6(0.2)	0.6(0.2)	0.5(0.2)	0.5(0.2)	0.5(0.2)	0.5(0.2)	0.5(0.1)	0.5(0.1)	0.5(0.1)	0.4(0.1)

O_3 was analyzed based on 8-hour maximum levels per time or day. SD, standard deviation; min, minimum; max, maximum; ppb, parts per billion; ppm, parts per million

Supplementary Table 2

Summary statistics for random sampling of urolithiasis cases with corresponding data on meteorological factors and ambient air pollutants in Korea between 2002 and 2017

		2002	2003	2004	2005	2006	2007	2008	2009	2010	2011	2012	2013	2014	2015	2016	2017
**Urolithiasis (*N* = 300,000)**		16,306	17,922	18,913	19,323	19,181	17,938	17,692	17,815	17,438	18,222	20,875	20,407	18,987	19,252	19,848	19,881
**Incidence**																	
Ureteral Stone (n = 150,000)		6,646	7,393	7,985	8,400	8,620	9,551	9,615	9,603	9,557	9,571	10,895	10,853	10,122	10,183	10,535	10,471
Renal Stone (n = 150,000)		9,660	10,529	10,928	10,923	10,561	8,387	8,077	8,212	7,881	8,651	9,980	9,554	8,865	9,069	9,313	9,410
**Gender**																	
Ureteral Stone																	
Male		3,996	4,430	4,847	5,223	5,267	5,909	5,878	6,033	5,899	5,877	6,809	6,741	6,365	6,327	6,582	6,634
Female		2,650	2,963	3,138	3,177	3,353	3,642	3,737	3,570	3,658	3,694	4,086	4,112	3,757	3,856	3,953	3,837
Renal Stone																	
Male		5,665	6,120	6,547	6,521	6,339	5,001	4,848	4,965	4,834	5,262	6,060	5,754	5,385	5,455	5,560	5,628
Female		3,995	4,409	4,381	4,402	4,222	3,386	3,229	3,247	3,047	3,389	3,920	3,800	3,480	3,614	3,753	3,782
**Age, Year**																	
Ureteral Stone	< 20	290	307	306	310	317	369	343	338	349	326	312	357	337	272	326	275
	20 - 60	5,452	6,040	6,574	6,925	7,013	7,577	7,580	7,500	7,434	7,421	8,263	8,252	7,510	7,574	7,601	7,486
	60 <	904	1,046	1,105	1,165	1,290	1,605	1,692	1,765	1,774	1,824	2,320	2,244	2,275	2,337	2,608	2,710
Renal Stone	< 20	323	352	338	332	336	222	204	162	202	190	236	184	173	150	145	152
	20 - 60	7,587	8,353	8,642	8,686	8,290	6,433	6,177	6,167	5,835	6,282	7,092	6,752	6,198	6,142	6,224	6,171
	60 <	1,750	1,824	1,948	1,905	1,935	1,732	1,696	1,883	1,844	2,179	2,652	2,618	2,494	2,777	2,944	3,087
**Meteorology (Mean, SD)**																	
Average Temperature (°C)		13.6(9.4)	14.0(8.8)	14.6(9.3)	14(10.3)	14.4(9.3)	14.9(9.0)	14.3(9.6)	14.0(9.5)	13.8(10.4)	13.4(10.3)	13.9(10.9)	14.4(10.6)	14.4(9.4)	14.6(9.4)	14.7(10.3)	14.2(10.4)
High Temperature (°C)		18.1(9.7)	18.4(9.0)	19.4(9.4)	18(10.4)	18.8(9.5)	19.2(9.0)	18.8(9.7)	18.6(9.7)	18.2(10.5)	17.8(10.3)	18.4(11.0)	18.9(10.7)	19.1(9.5)	19.4(9.6)	19.4(10.4)	19.1(10.3)
Low Temperature (°C)		9.7(9.5)	10.3(9.1)	10.6(9.5)	10(10.6)	10.6(9.6)	11.3(9.3)	10.5(9.7)	10.2(9.6)	10.2(10.7)	9.7(10.5)	10.1(11.1)	10.6(10.9)	10.4(9.8)	10.6(9.5)	10.7(10.5)	10.0(10.7)
Daily Range (°C)		8.4(3.1)	8.1(3.0)	8.7(3.0)	8.4(2.8)	8.1(2.9)	7.8(2.9)	8.3(2.9)	8.3(3.0)	8.0(3.0)	8.0(3.1)	8.2(2.8)	8.2(3.1)	8.7(3.2)	8.8(3.2)	8.7(2.9)	8.8(3.0)

Vapor Pressure (hPa)	12.0(7.9)	12.6(7.6)	12.6(8.1)	12.5(8.7)	12.6(8.1)	13.1(8.4)	12.2(8.0)	11.7(7.4)	12.7(8.9)	11.9(8.3)	12.2(8.5)	12.3(8.8)	13.0(8.2)	12.3(7.8)	12.9(8.8)	12.3(8.9)
Solar Radiation (MJ/m^2)	13.1(6.8)	12.0(6.7)	13.2(6.9)	13.6(7.0)	12.8(6.8)	12.8(6.7)	13.7(6.7)	13.9(7.1)	13.1(6.7)	13.2(6.9)	13.2(6.5)	13.0(6.8)	13.3(6.5)	13.1(6.6)	13.2(6.4)	13.7(6.9)
Sunshine Duration (hr)	12.1(1.7)	12.2(1.7)	12.2(1.7)	12.3(1.7)	12.2(1.7)	12.3(1.7)	12.2(1.7)	12.2(1.7)	12.2(1.7)	12.2(1.7)	12.3(1.7)	12.2(1.7)	12.2(1.7)	12.1(1.7)	12.2(1.7)	12.2(1.7)
Wind Speed (m/s)	2.5(1.2)	2.2(1.0)	2.4(0.9)	2.5(1.0)	2.4(1.0)	2.4(1.0)	2.4(0.9)	2.5(1.0)	2.5(1.0)	2.6(1.0)	2.7(1.0)	2.7(1.1)	2.5(1.0)	2.6(1.0)	2.4(1.0)	2.3(0.9)
Daily Rain (mm)	9.8(22.2)	12(24.6)	10(19.5)	9.6(19.2)	10(25.5)	7.9(14.2)	9.2(19.6)	11(26.1)	10(19.4)	13.1(32)	10.8(21)	10.2(20.3)	8.7(18.3)	6.1(10.6)	7.9(16.9)	6.2(16.3)
Dew Point Temperature (°C)	6.1(11.1)	7.3(10.5)	6.7(11.4)	5.9(12.7)	6.8(11.2)	7.6(10.8)	6.3(11.6)	5.9(11.3)	6.5(12.2)	5.2(12.4)	4.4(12.5)	6.6(12.1)	7.1(11.7)	6.8(10.6)	6.8(12.0)	5.8(12.3)
Humidity (%)	63(15.5)	66(15.1)	62(15.7)	61(15.9)	62(15.3)	64(15.0)	61(15.5)	61(15.9)	64(15.7)	61(17.2)	59(16.1)	63(16.1)	65(16.9)	65(16.2)	64(16.4)	61(16.1)
Daily snow (cm)	1.4(1.5)	2.2(2.6)	4.6(6.2)	3.0(4.9)	3.6(2.6)	1.8(3.9)	3.8(3.9)	2.4(2.4)	7.6(6.3)	2.7(2.0)	3.9(3.1)	5.9(4.3)	2.0(1.8)	2.4(2.5)	1.9(2.9)	1.9(1.6)
Cloud (1/10)	4.7(3.3)	5.3(3.2)	4.5(3.1)	4.5(3.2)	4.9(3.0)	5.2(3.1)	4.7(3.0)	4.6(3.1)	5.1(3.2)	5.0(3.3)	5.0(3.0)	4.8(3.1)	4.8(3.1)	4.8(3.1)	4.8(2.9)	4.6(3.0)
**Air Pollutants (Mean, SD)**																
$PM_{2.5}$ (ug/m^3)	-	-	-	-	-	-	-	-	-	-	28(12)	27(14)	25(14)	24(12)	25(14)	23(12)
PM_{10} (ug/m^3)	69(66)	60(32)	58(26)	58(27)	58(42)	58(36)	55(32)	52(28)	50(28)	49(31)	44(20)	47(23)	48(26)	47(34)	46(19)	45(20)
O_3 (100ppb)	1.6(0.8)	1.7(0.9)	1.7(0.9)	1.9(0.9)	1.8(0.9)	1.8(0.9)	2.0(0.9)	2.1(1.0)	1.9(0.9)	2.0(1.0)	2.1(1.0)	2.2(1.0)	2.3(1.1)	2.2(1.0)	2.4(1.1)	2.5(1.1)
NO_2 (100ppb)	3.2(1.1)	3.1(1.3)	3.2(1.2)	2.9(1.2)	3.0(1.2)	3.2(1.3)	3.2(1.4)	3.1(1.3)	3.2(1.3)	3.1(1.3)	3.1(1.2)	3.2(1.3)	3.1(1.3)	3.1(1.3)	2.9(1.1)	2.8(1.1)
SO_2 (100ppb)	0.5(0.2)	0.5(0.2)	0.6(0.2)	0.5(0.2)	0.5(0.2)	0.6(0.3)	0.6(0.2)	0.5(0.2)	0.5(0.2)	0.5(0.2)	0.5(0.2)	0.5(0.2)	0.5(0.2)	0.5(0.1)	0.4(0.1)	0.4(0.1)
CO (10ppm)	0.7(0.2)	0.6(0.2)	0.6(0.2)	0.6(0.2)	0.6(0.2)	0.6(0.2)	0.6(0.2)	0.6(0.2)	0.5(0.2)	0.5(0.2)	0.5(0.2)	0.5(0.2)	0.5(0.1)	0.5(0.1)	0.5(0.1)	0.4(0.1)

O_3 was analyzed based on 8-hour maximum levels per time or day.

SD, standard deviation; min, minimum; max, maximum; ppb, parts per billion; ppm, parts per million

Supplementary Table 3

Comparison of urolithiasis incidence rates among different age groups between 2002 and 2017

Age Group	Male			Female		
	Observation period (person-years)	No. of occurrence	Incidence rate (/1,000 person-year)	Observation period (person-years)	No. of occurrence	Incidence rate (/1,000 person-year)
0	225,242	68	0.30	212,210	32	0.15
1-4	916,978	1,162	1.27	864,654	436	0.50
5-9	1,243,294	2,391	1.92	1,151369	1,280	1.11
10-14	1,654,964	6,892	4.16	1,518,262	3,572	2.35
15-19	1,826,179	30,296	16.59	1,612,235	16,158	10.02
20-24	1,625,371	64,327	39.58	1,430,049	31,433	21.98
25-29	1,802,805	138,573	76.86	1,736,144	43,160	24.86
30-34	1,866,397	204,831	109.75	1,828,951	53,789	29.41
35-39	2,060,233	241,193	117.07	2,038,914	67,327	33.02
40-44	2,071,431	252,247	121.78	2,059,992	84,416	40.98
45-49	2,044,641	252,208	123.35	2,028,717	102,716	50.63
50-54	1,887,973	243,702	129.08	1,910,158	123,204	64.50
55-59	1,360,747	216,987	159.46	1,405,948	119,970	85.33
60-64	1,057,035	173,444	164.08	1,125,201	91,754	81.54
65-69	833,242	132,316	158.80	978,926	69,451	70.95
70-74	672,894	90,280	134.17	893,120	47,549	53.24
75-79	410,726	50,129	122.05	673,641	28,188	41.84
80-84	186,008	22,411	120.48	409,501	12,967	31.66
≥85	94,736	9,479	100.06	271,873	5,886	21.65

Supplementary Table 4. Akaike information criterion for the association between daily medical care utilization for urolithiasis and selected meteorological factors and ambient air pollutants (MFAPs)

Test	MFAP as effect	DF	AIC
1	Average Temperature, Diurnal Temperature Range, Dew point Temperature, Humidity, Sunshine Duration, Solar Radiation, $PM_{2.5}$, PM_{10}, CO, NO_2, SO_2, O_3	12	44.63
2	Average Temperature, Diurnal Temperature Range, Dew point Temperature, Humidity, Sunshine Duration, Solar Radiation, $PM_{2.5}$, PM_{10}, CO, NO_2, SO_2	11	38.53
3	Average Temperature, Diurnal Temperature Range, Dew point Temperature, Humidity, Sunshine Duration, Solar Radiation, $PM_{2.5}$, PM_{10}, CO, NO_2	10	35.73
4	Average Temperature, Diurnal Temperature Range, Dew point Temperature, Humidity, Sunshine Duration, Solar Radiation, $PM_{2.5}$, PM_{10}, CO	9	28.17
5	Average Temperature, Diurnal Temperature Range, Dew point	8	33.00

	Temperature, Humidity, Sunshine Duration, Solar Radiation, $PM_{2.5}$, PM_{10}		
6	Average Temperature, Diurnal Temperature Range, Dew point Temperature, Humidity, Sunshine Duration, Solar Radiation, $PM_{2.5}$	7	22.77
7	Average Temperature, Diurnal Temperature Range, Dew point Temperature, Humidity, Sunshine Duration, Solar Radiation, $PM_{2.5}$, CO	8	18.13
8	Average Temperature, Diurnal Temperature Range, Dew point Temperature, Humidity, Sunshine Duration, $PM_{2.5}$, CO	7	12.18
9	Average Temperature, Diurnal Temperature Range, Dew point Temperature, Sunshine Duration, $PM_{2.5}$, CO	6	8.56
**10**	**Average Temperature, Diurnal Temperature Range, Sunshine Duration, $PM_{2.5}$, CO**	**5**	**4.89**
11	Average Temperature, Diurnal Temperature Range, Sunshine Duration, $PM_{2.5}$, PM_{10}	5	19.67

12	Average Temperature, Diurnal Temperature Range, Sunshine Duration, $PM_{2.5}$, SO_2	5	12.65
13	Average Temperature, Diurnal Temperature Range, Sunshine Duration, $PM_{2.5}$, NO_2	5	17.47
14	Average Temperature, Diurnal Temperature Range, Sunshine Duration, $PM_{2.5}$, O_3	5	15.90

MFAPs, meteorological factors and ambient air pollutants; DF, degree of freedom ; AIC, Akaike Information Criterion ; $PM_{2.5}$, particulate matter ≤2.5 μm; PM_{10}, PM ≤10 μm in diameter; CO, carbon monoxide; NO_2, Nitrogen Dioxide; SO_2, Sulfur Dioxide; O_3, Ozone

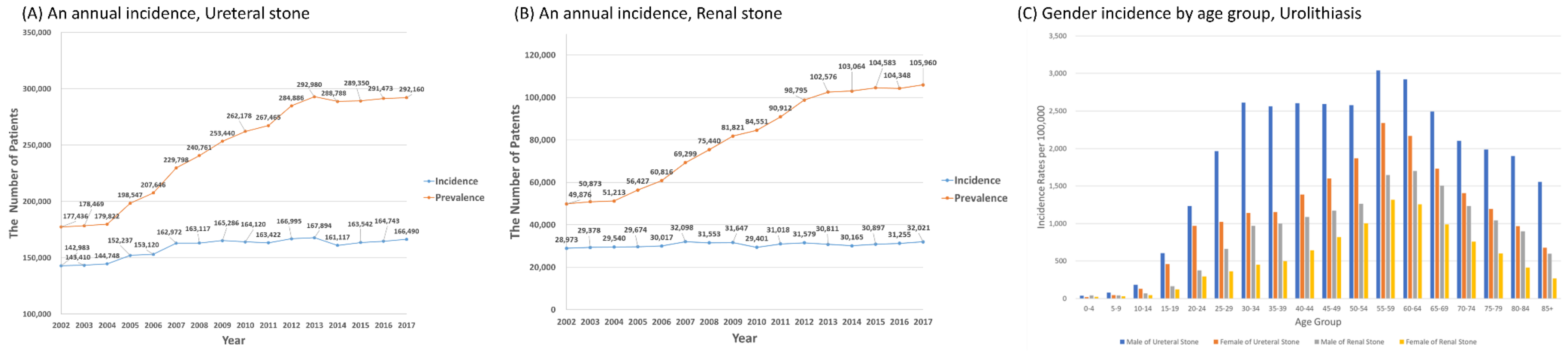

Supplementary Figure 1. Prevalence and incidence of (a) ureteral and (b) renal stones between 2002 and 2017. (c) Histogram of the sex-dependent incidence of urolithiasis according to age group.

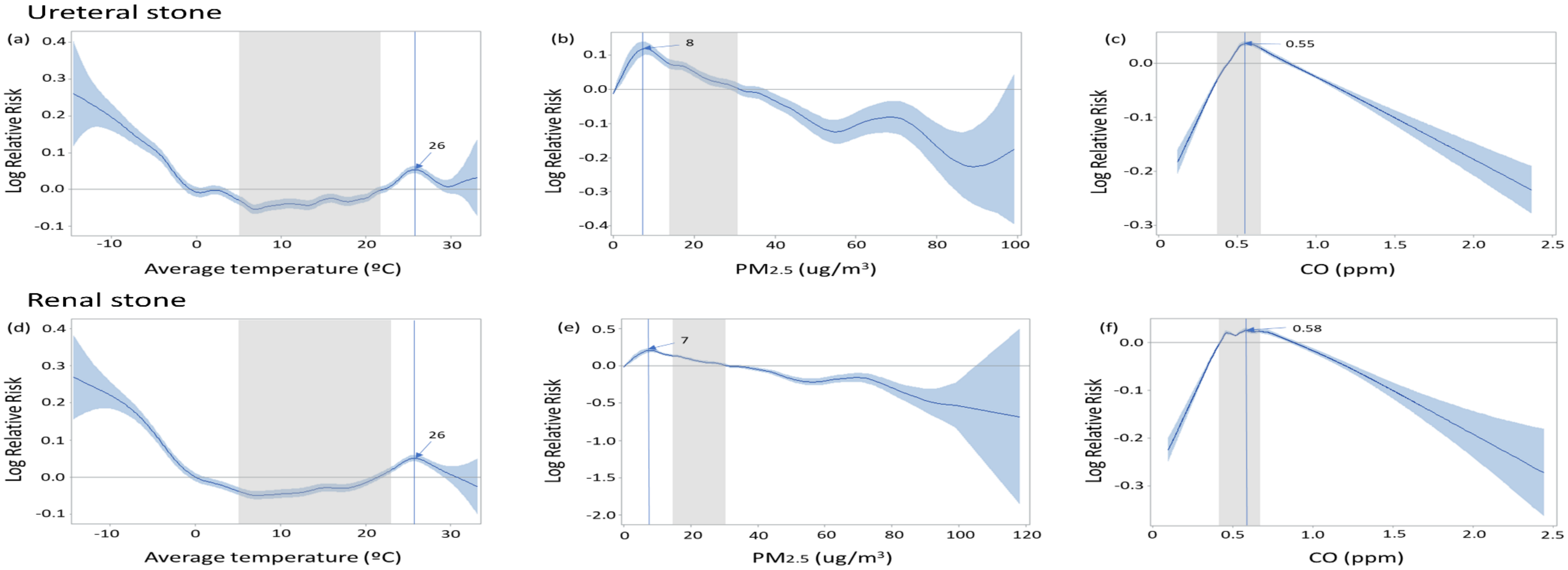

Supplementary Figure 2

Generalized additive model with natural splines for the effect of selected MFAPs on the number of medical care utilization for urolithiasis. Ureteral stones: (a) average temperature, (b) $PM_{2.5}$, and (c) CO. Renal stones: (d) average temperature, (e) $PM_{2.5}$, and (f) CO. The bold line indicates the relative effect sizes for urolithiasis, and the blue area indicates 95% confidence intervals. The X- and Y-axes represent the selected MFAPs and relative effect sizes for urolithiasis, respectively. MFAPs, meteorological factors and ambient air pollutants; $PM_{2.5}$, particulate matter $\leq$2.5 µm; CO, carbon monoxide.
